# Factors influencing performance of community-based health volunteers’ activities in the Kassena-Nankana Districts of Northern Ghana

**DOI:** 10.1371/journal.pone.0212166

**Published:** 2019-02-20

**Authors:** Samuel Chatio, Paul Welaga, Philip Teg-Nefaah Tabong, Patricia Akweongo

**Affiliations:** 1 Navrongo Health Research Centre, Navrongo, Ghana; 2 University of Ghana, School of Public Health, Accra, Ghana; Makerere University College of Humanities and Social Sciences, UGANDA

## Abstract

**Background:**

An increasing demand for health care services and getting health care closer to doorsteps of communities has made health managers to use trained community-based health volunteers to support in providing health services to people in rural communities. Community volunteerism in Ghana has been identified as an effective strategy in the implementation of Primary Health Care activities since 1970s. However, little is known about the performance of these volunteers engaged in health interventions activities at the community level. This study assessed the level of performance and factors that affect the performance of health volunteers’ activities in Northern Ghana.

**Methods:**

This was a cross-sectional study using quantitative method of data collection. Two hundred structured interviews were conducted with health volunteers. Data collectors visited health volunteers at home and conducted the interviews after informed consent was obtained. STATA Version 11.2 was used to analyze the data. Descriptive statistics were used to assess the level of performance of the health volunteers. Multiple logistic regression models were then used to assess factors that influence the performance of health volunteers.

**Results:**

About 45% of volunteers scored high on performance. In the multivariate analysis, educational status [OR = 4.64 95% CI (1.22–17.45)] and ethnicity [OR = 1.85 95% CI (1.00–3.41)] were the factors that influenced the performance of health volunteers. Other intermediary factors such as incentives and means of transport also affected the performance of health volunteers engaged in health intervention activities at the community level.

**Conclusion:**

The results suggest that higher educational status of health volunteers is more likely to increase their performance. In addition, providing non-monetary incentives and logistics such as bicycles, raincoats, torch lights and wellington boots will enhance the performance of health volunteers and also motivate them to continue to provide health services to their own people at the community level.

## Introduction

An increasing demand for health care services has made health service managers resort to seeking greater collaboration with communities and the use of trained Community-Based Health Volunteers (CBHVs) to support in providing health services to people at the community level [1]. These health volunteers may have one or more functions to carry out depending on the roles and responsibilities assigned to them by health managers. In many settings, community-based health volunteers are selected by their own community members [2] and trained by health professionals to support formal health workers to provide basic health services to their own community people [3, 4].

It is demonstrated that CBHVs involvement in health care especially maternal and child health services has helped to reduce morbidity and mortality in certain communities [5, 6]. Similarly, with the introduction of Integrated Management of Childhood Illnesses (IMCI), community-based health volunteers have played an important role in helping to prevent diseases and also promote healthy behaviors at the community level [3, 7]. Furthermore, health volunteers’ activities have had positive impact on child vaccination, increase level of child growth monitoring, and increase in provision of iron tablets to pregnant women [5]

Nonetheless, there are factors that affect the performance of community based-health volunteers. It is revealed that CBHVs are not able to work very well in situations where they are not getting the needed support from community members [8]. In addition, lack of effective supervision and incentives negatively affect the performance of health volunteers [8]. Unreliable medical supplies, inadequate training and workload have also been reported to have negative effect on the performance of health volunteers [5]. The level of education has also been reported as one of the factors affecting the performance of community-based health volunteers [5, 8]. Evidence exist that community-directed health interventions mostly fail because of lack of funds to motivate CBHVs engaged in health care delivery activities at the community level [9]. Volunteerism has been labeled as people offering services without being paid. However, there is a growing debate as to whether health volunteers should be given allowances or non-monetary incentives such as bicycles and other logistics to enhance their performance [9].

Ghana recognized community involvement and the volunteerism concept in the 1970s as a key development goal [10, 11]. Community volunteerism was identified as a major and effective component in the implementation of Primary Health Care (PHC) activities. The idea was part of the government’s plan to make health care services available at the doorsteps of people through community participation and volunteerism [10, 11]. Therefore, health volunteers were selected by their own community members, trained by health managers and brought back and introduced to Chiefs, elders and other community members during community durbars. Their primary responsibility has been to help formal health workers to provide basic health care services to all people in their respective communities [7, 11]. The basic criteria for selecting health volunteers include age, being a member or leader of existing social groups and networks in the community, have proven record of active participation in communal work at the community, having a stable voluntarism character, trustworthiness, long term residence in the community and ready to work under the supervision of the community leaders and the sub district health managers [3, 12]. Since the introduction of community-based health volunteer concept, Kassena-Nankana East and Kassena-Nankana West districts have been using community-based health volunteers to carry out health intervention activities in the communities. Among the intervention programmes volunteers are involved in the study districts include Polio immunization, Health education campaigns, Nutrition, Case identification and reporting, Integrated Management of Childhood Illness (IMCI), Immunization programmes, Elephantiasis drugs distribution, Cerebro-Spinal Meningitis (CSM) programme among others [10, 13]. Despite several years of health volunteers’ involvement in health intervention activities in Ghana, little is known on factors affecting their performance. This study therefore assessed the level of performance and factors affecting the performance of community-based health volunteers in the Kassena-Nankana East and West Districts in the Upper East Region of Northern Ghana.

## Methodology

### Ethical considerations

Ethical approval for the study was obtained from the Ghana Health Service ethics committee. Approval was also sought at the Regional Health Directorate and at the district health management offices in the study districts before data collection started. Written informed consent was obtained from all study participants before they were interviewed. Study participants were informed about the purpose of the study, how they were selected to take part in the study, their right to refuse being interviewed and the right to withdraw from the study in the process of the interview. Study participants were also assured of the confidentiality of the information given.

### Study design

This was a follow-up cross-sectional quantitative study to a qualitative research on retention and sustainability of community-based health volunteer’ activities in rural Northern Ghana (https://doi.org/10.1371/journal.pone.0174002). The quantitative data were used to describe the background characteristics of the study participants, the level of performance and also assess factors that influence the performance of community-based health volunteers. The performance of health volunteers in this study was categorized into two groups; high or low. This was done based on the responses to the questions on the activities health volunteers were expected to undertake in the communities. These activities included health education, submission of reports, attending meetings and taking part in immunization activities at the community level. The performance of a volunteer was therefore measured as high if he/she performed all the four activities from the responses to the questions, and low if he/she failed to perform at least one of the activities.

### Study area

The study was conducted in the Kassena-Nankana East District (KNED) and Kassena-Nankana West District (KNWD) of Northern Ghana. The districts cover an area of 1,675 square kilometres with an estimated population of about 153,000 under surveillance by the Navrongo Health and Demographic Surveillance System (NHDSS) [14], operating under the Navrongo Health Research Centre (NHRC). The population is predominantly rural with subsistence farming as the mainstay of the districts’ economy. The District have two distinct seasons, a raining season that runs from May to September and a long dry season from October to April with hardly any rains. Most of the population lives in rural and sparsely scattered settlements and this makes health service delivery very difficult. There are two main ethnic groups in the area, the Kassem and the Nankani speaking people.

The districts have 6 health centers, 2 clinics, and 28 functional Community-based Health Planning and Services (CHPS) compounds located in various villages with resident Community Health Officers (CHOs) offering doorstep health services to the people [15,16]. The main referral hospital (War Memorial Hospital) is located at the capital (Navrongo Town) of the KNED. There are Community-Based Health Volunteers in the communities providing basic health services to community members through the supervision of professional health workers.

### Sampling strategy and sample size

**Fig 1:**
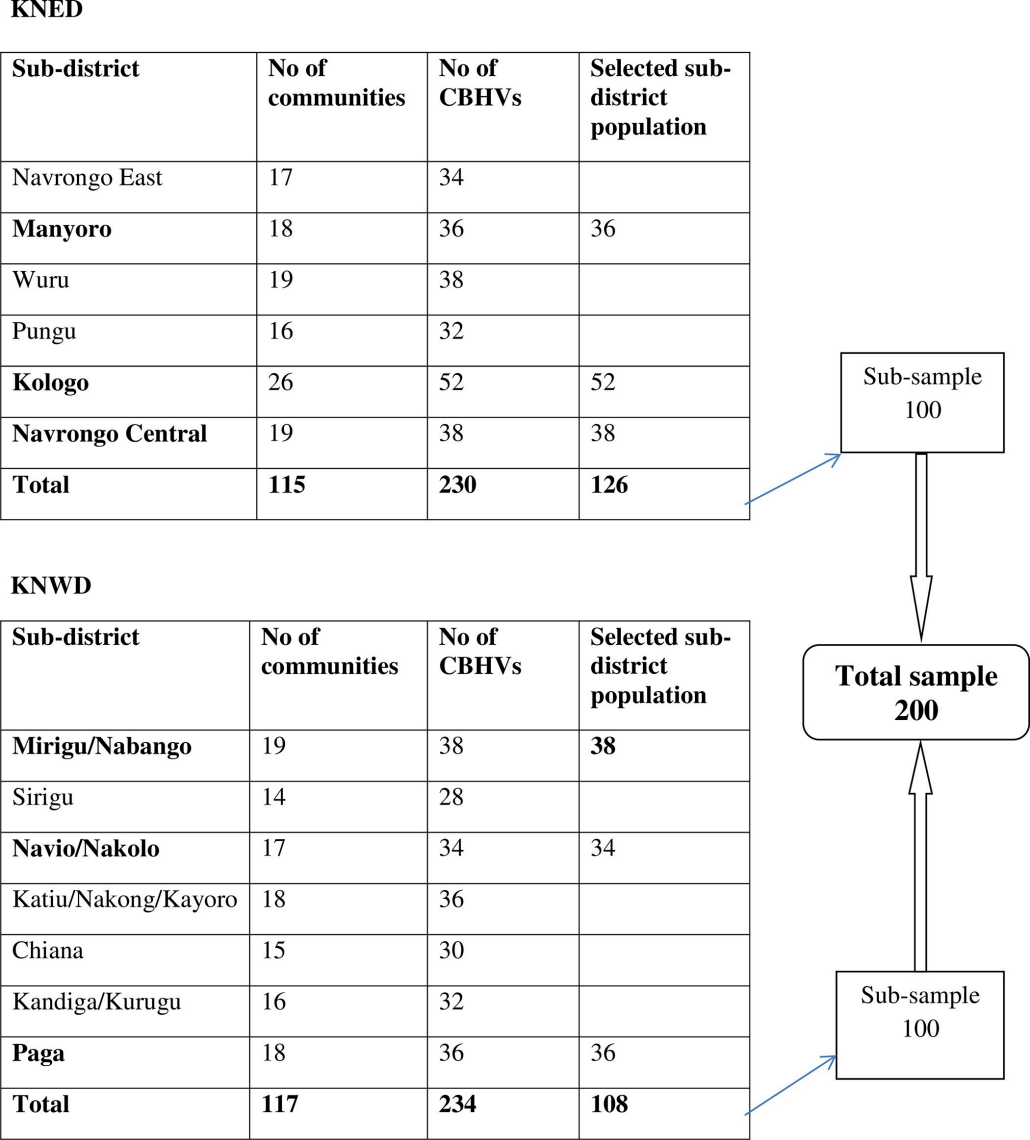
Sampling strategy.

The two districts have been divided into sub-districts and each sub-district has a number of communities with two health volunteers working in each community. The total number of communities per sub-district was compiled with the number of health volunteers in each community. KNED has a total of six sub-districts, one hundred and fifteen (115) communities with two hundred and thirty (230) community health volunteers. KNWD on the other hand has a total of seven (7) sub-districts, one hundred and seventeen communities (117) with two hundred and thirty-four (234) community health volunteers (Fig 1) [17]. The study covered all the sub-districts in the two districts.

In calculating the sample size, we used the normal approximation to the hyper geometric distribution because of the small population of health volunteers we were sampling from. With an estimated total population of 464 health volunteers in the study area, assuming a 70% retention rate of health volunteers with an error margin of 5%, and using a 95% confidence interval gave an estimated sample size of 191. In order to take care of missing data, the sample size was increased to 200. Comparing the total number of health volunteers in the two districts, the difference was small. Therefore, since the sample size for the study was two hundred, one hundred health volunteers were interviewed in each district. Simple random sampling technique was used to select three sub-districts each in the two districts. The sub-districts selected in the KNED were Manyoro, Kologo and Navrongo central while Mirigu, Navio/Nakolo and Paga were selected in the KNWD (Fig 1). Health volunteers in the sub-districts selected were contacted and volunteers who were available at the time of the study were interviewed to arrive at the sample of 200 participants. All participants consented to take part in the study.

### Data collection techniques

A structured survey questionnaire was used to interview 200 CBHVs engaged in health intervention activities in the study area. The data collectors visited health volunteers at home and conducted the interviews after informed consent was obtained.

### Training of data collectors

Two senior secondary school graduates were recruited and trained for data collection. The training covered areas such as the purpose and objectives of the study and data collection techniques. Data collectors were also taken through the study questionnaire first in English and in the two main local languages spoken in the study area (Kassem and Nankam). To help the data collectors to have a better understanding of the questionnaire and to ask the questions appropriately, role-play was done during the training. The questionnaire was pre-tested at the end of the training where twenty volunteers in a community in Bolgatanga Municipal were recruited for the pre-testing exercise. This was done to enable the study team to finalize the questionnaire before the actual data collection and also to enable the data collectors to ask the questions appropriately in the local languages during data collection.

At each stage of the data collection, the work of the data collectors was supervised to make sure that the data collection was done accurately. Study participants were asked questions on the activities they were involved in as health volunteers and factors affecting their work or performance. All completed questionnaires were checked and submitted for data entry.

### Data processing and analysis

The data were double-entered and verified using Epidata 3.1 with built in consistency checks to control data input. Data cleaning by way of checking for consistencies among variables were carried out by running frequencies and cross tabulations using STATA Version 11.2. Descriptive analyses were used to present socio-demographic characteristics of respondents. The statistical point estimates were computed and presented as means, proportions or percentages for all the background characteristics and factors affecting performance. The performance of volunteers in this study was categorized into two groups; high or low. The categorization was done based on the responses to the questions on the activities health volunteers were expected to undertake in the communities. These activities were grouped into four and included health education, submission of reports, attending meetings and taking part in immunization activities by helping to organize mothers for weighing at the community level. The performance of a volunteer was therefore categorized as high if he/she performed all the four activities from the responses to the questions, and low if he/she failed to perform at least one of the activities.

Unadjusted and adjusted odds ratios with 95% confidence intervals were computed to assess the relationship between the performance of community volunteers and selected variables using bivariate and multiple logistic regression models. The independent variables included in our analysis were age, sex, educational status, religion, ethnicity, marital status and occupation. All these variables were included in the multiple logistic regression model. The selection of the independent variables was based on literature from previous studies and observations. Reference categories were defined as those usually associated with the lowest performance. All variables with P-values <0.10 were included in the multiple logistic regression model, and adjusted odds ratios with 95% confidence intervals were obtained. Intermediate factors affecting volunteers’ performance were also explored using logistic regression. Significance level was set at 5%.

## Results

### Socio demographic characteristics of health volunteers

The results showed that 53% (106) of the health volunteers in this study were males. About 69% (137) of them were thirty five years and above. Majority 60% (120) of the volunteers completed primary/middle/Junior school education. The mean age of the participants was 42 years (s.d = 12). Most of the volunteers 70.3% (153) were Christians. Most of them 76.5% (153) were married/living together. Also, about 50% (100) of the volunteers’ main occupation was farming while 16.5% (33) of them indicated that the health voluntary work was their main occupation. Majority of the volunteers who took part in this study 57.5% (115) were Kassem speaking people (Table 1).

**Table 1:** Background characteristics of volunteers.

Variable	n(%)	Number with high performance n(%)	P-value
**Age of respondents**			
17–24	16(8.0)	8(50.0)	
25–34	47(23.5)	27(57.5)	0.110
35+	137(68.5)	55(40.2)	
**Sex**			
Male	106(53.0)	57(53.8)	0.008
Female	94(47.0)	33(35.1)	
**Educational status**			
No education	18(9.0)	4(22.2)	
Primary/Middle/JSS	120(60.0)	48(40.0)	
Secondary/Tertiary/Higher	62(31.0)	38(61.3)	0.003
**Religion**			
Traditional	41(23.6)	15(36.6)	
Christian	153(70.3)	72(47.4)	0.456
Muslim	6(6.1)	3(50.0)	
**Ethnicity**			
Kassem	115(57.5)	42(36.5)	
Nankam	85(42.5)	48(56.5)	0.005
**Marital status**			
Never married	21(10.5)	13(61.9)	
Married/living together	153(76.5)	69(45.1)	0.103
Widowed/Divorced	26(13.0)	8(30.8)	
**Occupation**			
Volunteer	33(16.5)	14(42.4)	
Trader/Housewife	53(26.5)	19(35.9)	
Civil servant	14(7.0)	11(78.5)	0.040
Farming	100(50.0)	46(46.0)	

### Factors affecting performance of health volunteers

Performance of CBHVs was conceived in terms of the number of times a volunteer attended meetings, submitted reports, participated in immunization activities such as organizing mothers for weighing and providing health education to community members. The results showed that majority of the volunteers 77.4% (154) always attended meetings, 77.5% (155) always submitted reports to their supervisors and 67.3% (134) always took part in immunization activities at the community level whilst 86.5% (173) carried out health education.

The overall performance of the volunteers was assessed on the combination with volunteers’ ability to always attend meetings, always submit reports to their supervisors, always taking part in immunization activities by organizing mothers for weighing at the community level and always providing health education to community members. Volunteers who reported always taking part in these four activities had a high performance rating whilst volunteers who reported that they somehow took part in at least one of the activities were considered low performing volunteers. The results showed that 45% (90) of volunteers scored high on performance whilst 55% (110) of the volunteers scored low on performance.

Furthermore, our results showed that sex (P = 0.009), educational status (P = 0.004) and ethnicity (P = 0.005) were factors that influenced performance of health volunteers in bivariate analysis. After adjusting for the effect of other confounding variables in the multiple logistic regression model, volunteers with secondary or higher education had fourfold increased odds of performing well compared to those with no education, [OR = 4.64 95% CI (1.22–17.45)]. Volunteers from Nankam ethnic group had 85% increased odds of performing well compared to the Kassem ethnic group, [OR = 1.85 95% CI (1.00–3.41)] (Table 2).

**Table 2:** Factors affecting the performance of community-based health volunteers.

		Bivariate (Unadjusted)		Multivariate (Adjusted)	
Variable	n (%)	OR (95% CI)	Overall p-value	OR(95%.CI)	Overall p-value
**Age**					
17–24	16(8.0)	1			
25–34	47(23.5)	1.35(0.43–4.21)	0.114		
35+	137(68.5)	0.67(0.24–1.89)			
**Sex**					
Male	106(53.0)	2.15(1.22–3.80)	0.009	1.56(0.81–3.00)	0.187
Female	94(47.0)	1			
**Educational status**					
No education	18(9.0)	1		1	
Primary/JSS	120(60.0)	2.33(0.72–7.52)	0.004	2.05(0.60–7.00)	
Secondary/tertiary	62(31.0)	5.54(1.63–18.83)		4.64(1.22–17.45)	0.029
**Religion**					
Traditional	41(20.6)	1			
Christian	152(76.4)	1.56(0.77–3.18)	0.459		
Muslim	6(3.0)	0.31–9.70			
**Ethnicity**					
Kassem	115(57.5)	1		1	
Nankam	85(42.5)	2.25(1.27–4.00)	0.005	1.85(1.00–3.41)	0.049
**Marital status**					
Never married	21(10.5)	1			
Married	153(76.5)	0.51(0.20–1.29)	0.111		
Widowed/divorced	26 (13.0)	0.27(0.08–0.92)			
**Main occupation**					
CBHV	33(16.5)	1			
Trader/housewife	53(26.5)	0.76(0.31–1.85)		1.34(0.50–3.59)	
Civil servant	14(7.0)	4.98(1.17–21.24)	0.066	6.24(1.39–28.26)	0.113
Farming	100(50.0)	1.16(0.52–2.56)		1.63(0.66–4.01)	

### Intermediary factors that affect the performance of CBHVs

**Fig 2:**
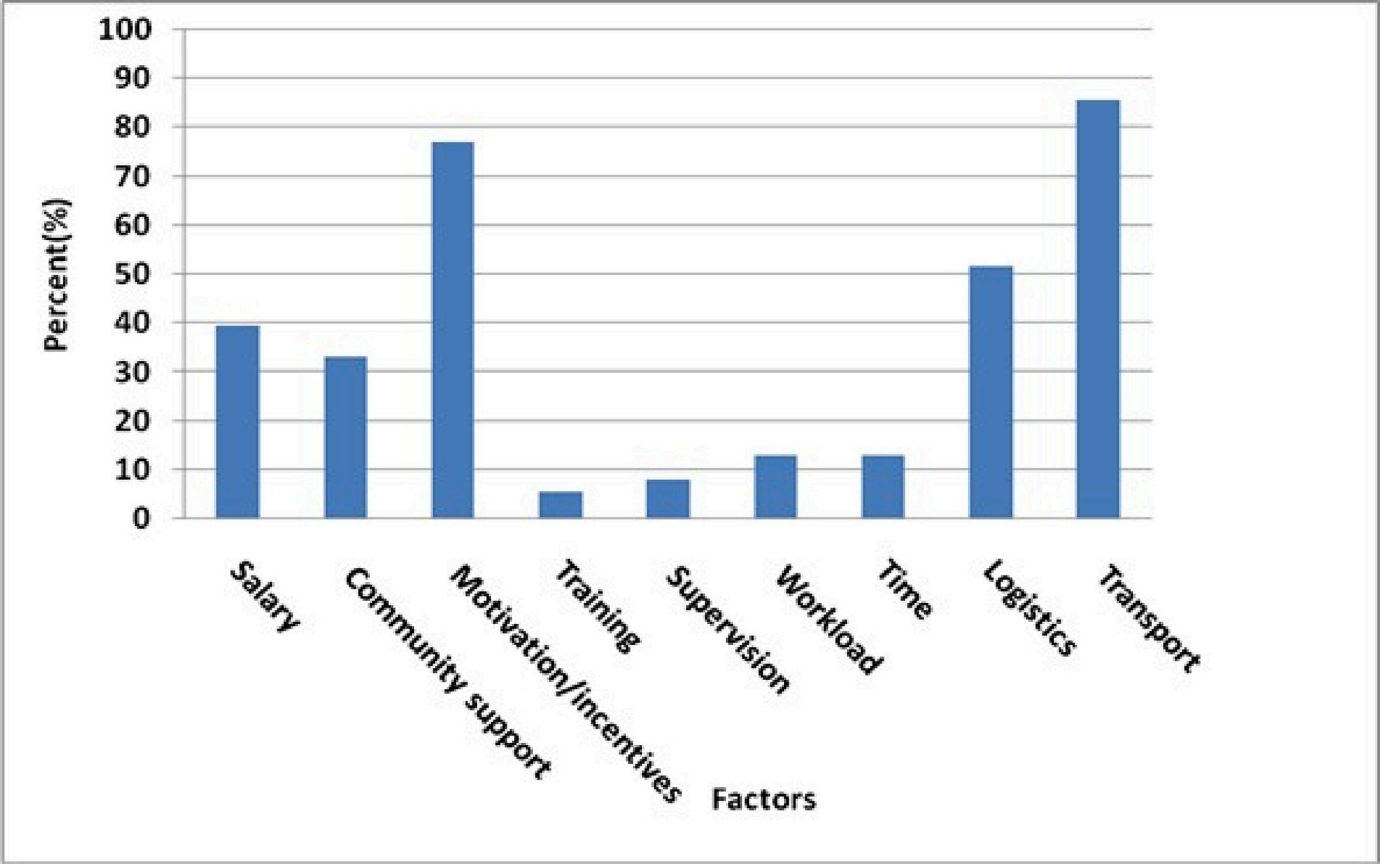
Intermediary factors affecting performance of health volunteers.

Fig 2 describes the percentage distribution of other intermediary factors reported by health volunteers affecting their performance. From the results, 85.5% (171) of the volunteers reported that lack of means of transport in the form of bicycles affected their performance. Seventy seven percent (154) of them reported lack of motivation/incentives while 51.5% (103) of volunteers said that lack of logistics affected their performance (Fig 2).

Other factors reported by CBHVs in the results were non-payment of salary, lack of community support, workload, no time, inadequate training and lack of supervision. However, less than 50% of health volunteers mentioned them as factors affecting their performance. A bivariate analysis was used to test the significance of these intermediary factors affecting volunteers’ performance. The findings showed that lack of means of transport as an intermediary factor had a negative effect on health volunteers’ performance [OR = 3.70 95% CI (1.44–9.54)].

## Discussion

It is demonstrated that the work of health volunteers and their performance is key as health volunteers’ involvement in maternal and child health services such as growth promotion and vaccination of children helps to improve the health of these children [11, 18]. It is revealed that the activities of health volunteers have also contributed significantly in access to health care and the use of health services such as immunization, weighing and health education and first aid medicines at the community level [7, 19]. However, based on the interpretation of our data, we found that Socio-demographic factors such as educational status and ethnicity were significantly associated with performance of health volunteers in providing health services at the community level. Volunteers with secondary or higher education were more likely to perform better than those without education or with primary education. This is consistent with earlier studies that reported that the level of education greatly influenced performance of health volunteers [5, 8].

Also, in our study, ethnicity has been found as one of the factors affecting the performance of health volunteers engaged in providing health services at the community level. Our findings revealed that the Nankani speaking volunteers were more likely to perform better than the Kassem speaking volunteers. However, the reason why the Nankani speaking volunteers could perform better than the Kassem speaking health volunteers was not established in the findings of our study.

Other factors such as workload, ineffective supervision, lack of community support, recognition, inadequate training and monetary incentives have been reported by earlier studies to have had a significant influence on the performance of health volunteers [5, 8]. Evidence also exist that distrust as a result of how volunteers were selected and lack of community support affected the morale and performance of health volunteers engaged in health activities at the community level [2]. It has also been reported that negative attitude of some professional health workers and community members towards health volunteers negatively affected health volunteers’ performance [20]. Nonetheless, this is not consistent with our study findings. These factors have been described in our study as intermediary factors, which were not found to be significant in the results. However, our study findings showed that only means of transport as an intermediary factor significantly influenced the performance of health volunteers. This was expected because without means of transport, the volunteers would not be able to freely move round the communities and do their work effectively as reported by some of the health volunteers in this study. It is demonstrated that lack of means of transport affected the ability of health volunteers to submit their reports to health workers [21]. Therefore, the findings of our study suggest that when health volunteers are provided with means of transport, it will greatly enhance their performance.

Also, a qualitative study conducted in the area reported that lack of other non-monetary incentives and logistics such as raincoats, torch lights, wellington boots and means of transport greatly influenced the performance of community-based health volunteers [17]. Lack of these non-monetary incentives discouraged health volunteers and thereby affecting their performance. When health volunteers are given these non-monetary incentives including awards to hard working volunteers, it would motivate them to work [17].

## Limitations

One limitation of our study is that we did not collect data to analyse the perspectives of health managers and supervisors of health volunteers on the challenges volunteers face in carrying out their activities. We hope future studies will be conducted to address this issue. In addition, we did not collect qualitative data on factors affecting volunteers’ performance to help us provide in-depth information on some of the issues that came up in the quantitative results.

## Conclusion

The importance of health volunteers in health service delivery at the community level cannot be ignored. Good education enhances the performance of health volunteers. Based on our interpretation of this data, engaging volunteers with secondary or higher education could improve their performance. Means of transport as intermediary factor has been reported to have significant influence on the performance of health volunteers. Based on the interpretation of our data, monetary incentives and other factors such as salary, workload and supervision do not directly affect the performance of volunteers engage in health intervention activities at the community level. Nonetheless, our recommendation is that when volunteers are provided with means of transport such as bicycles and other non-monetary incentives and logistics such as raincoats, torch lights and wellington boots, it will enhance their performance and also motivate them to continue to provide health care services to their own people at the community level.

## Supporting information

S1 File Study questionnaire. (DOCX)
