# Supplementary material for: Factors influencing performance of community-based health volunteers’ activities in the Kassena-Nankana Districts of Northern Ghana
Source: PLoS One. 2019 Feb 20;14(2):e0212166. doi: 10.1371/journal.pone.0212166 (PMC6382126; doi:10.1371/journal.pone.0212166)
Supplement: S1 File — (DOCX) [file pone.0212166.s001.docx]

| **SURVEY QUESTIONNAIRE**  **FACTORS INFLUENCING SUSTAINABILITY OF COMMUNITY-BASED HEALTH**  **VOLUNTEERS ACTIVITIES IN THE KNED AND KNWD** **SECTION 1: IDENTIFICATION** Name of interviewee________________________________________________________ RNAME | | | | | | | |
| --- | --- | --- | --- | --- | --- | --- | --- |
| COMPOUND NAME/ID. | |  |  |  |  |  | CMPNAID |
| DATE OF INTERVIEW |  |  |  |  |  |  | DAINT |
| FIELDWORKER CODE | | | | |  |  | FWCODE |
| FIELD SUPERVISOR CODE | | | | |  |  | FSCODE |
| RESULT OF INTERVIEW: | | | | |  |  | RESULT |
| COMPLETE, INTERVIEW | | | | |  | 1 |  |
| INCOMPLETE, REFUSED | | | | |  | 2 |  |
| INCOMPLETE, OTHER__________________________________________  (SPECIFY) | | | | |  | 3 |  |

**SECTION 2: BACKGROUND CHARACTERISTICS OF RESPONDENTS**

| **No** | **Questions and filters** | **Coding Categories** | **Skip to ≠** |
| --- | --- | --- | --- |
| 1. | How old are you now? | \|  \|  \| \| --- \| --- \| | Q1AGE |
| 2. | Sex of volunteer | Male……………………………………1  Female………………………………….2 | Q2SEX |
| 3. | Have you ever attended school? | Yes..........................................................1  No...........................................................2 | Q3EVESCH |
| 4. | What is the highest level of school you attended? | Primary...................................................1  Middle/JSS.............................................2  Secondary/SSS…....................................3  Tertiary/.higher......................................4  Other (specify)........................................5 | Q4LEVEL |
| 5. | What is your religion? | Traditional...............................................1  Christian...................................................2  Muslim.....................................................3  Other (specify).........................................4 | Q5RELIG |
| 6. | What is your ethnicity? | Kasem......................................................1  Nankam.....................................................2  Buli..........................................................3  Other (specify).........................................4 | Q6ETHNIC |
| 7. | What is your marital status now? | Never married...........................................1  Married.....................................................2  Living together.........................................3  Devoiced..................................................4  Widowed..................................................5  Separated..................................................6  Other (specify).........................................7 | Q7MARIT |
| 8. | What is your main occupation? | CBHV.......................................................1  Trader.......................................................2  Housewife................................................3  Civil servant.............................................4  Farming……….........................................5  Other (specify)………………………….6 | Q8OCCUPA |

**SECTION 3: ACTIVITIES OF CBHVs AND ATTRACTION**

| 9. | How long have you been working as a health volunteer in this community?  **Write 00 if less than one year** | \|  \|  \| \| --- \| --- \| | Q9WKLONG |
| --- | --- | --- | --- | --- | --- |
| 10 | What health intervention programs are you involved in this community? | 1_______________________________  2_______________________________  3_______________________________ | Q10HPROG |
| 11. | What exactly do you do as a health volunteer in this community?  **CIRCLE ALL THAT APPLY** | **Yes No**  Health education/talk..................1 2  Mobilizing mothers for  immunization...............................1 2  Weighing children…..………….1 2  Provide counseling services…....1 2  Defaulter tracing/follow up.........1 2  Provide first aid..........................1 2  Distribute polio drugs.................1 2  Treatment of minor illnesses.......1 2  Case identification and  reporting/referral.........................1 2  Other…………………………...1 2  (specify)...................................................... | Q11HEDU  Q11IMMU  Q11WEIGH  Q11COUNS  Q11FOLLO  Q11FAID  Q11PDRUG  Q11MILL  Q11CREPO  Q11OTHER  Q11SPECI |
| 12. | What motivated you to work as a health volunteer?  **CIRCLE ALL THAT APPLY** | **Yes No**  Help com. Members/sick people......1 2  To earn income.................................1 2  Prestige and respect..........................1 2  Seen as doctor in community ...........1 2  Enjoy working as health volunteer…1 2  Help get paid job in future………….1 2  Use idle time………………………..1 2  Other ……………………………….1 2  (specify)......................................................... | Q12HSICK  Q12INCOM  Q12PREST  Q12SDOC  Q12ENJOY  Q12PJOB  Q12UTIME  Q12OTHER  Q12SPECI |
| 13. | What will make you/somebody refuse to work as a health volunteer?  **CIRCLE ALL THAT APPLY** | **Yes No**  No salary..........................................1 2  Workload/Difficult..........................1 2  No respect by comm. members........1 2  No support by superiors ..................1 2  No support by community...............1 2  Spouse/family members refusal…..1 2  No effective supervision…………..1 2  No time………………………...….1 2  Other……………………………...1 2  (specify)…………………............................. | Q13NSALA  Q13WLOAD  Q13NRESP  Q13NSSUP  Q13NSCOM  Q13FREFU  Q13NESUP  Q13NTIME  Q13OTHER  Q13SPECI |
| 14. | What will make you/somebody agree to work as a volunteer and later dropout?  **CIRCLE ALL THAT APPLY** | **Yes No**  No salary..........................................1 2  Workload/Difficulty........................1 2  No respect by community................1 2  No support by superiors..................1 2  No support by comm. members…...1 2  Spouse/family members refusal…...1 2  No effective supervision…………...1 2  Old Age…………………………….1 2  No Motivation/Incentives………..…1 2  No time…………………………….1 2  Got job elsewhere...………………..1 2  Other……………………………….1 2  (specify)…………………………………… | Q14NSALA  Q14WLOAD  Q14NRESP  Q14NSSUP  Q14NSCOM  Q14FREFU  Q14NESUP  Q14OAGE  Q14NTIME  Q14GJOB  Q14OTHER  Q14SPECI |
| 15. | What do you think should be done to attract people to accept to work as health volunteers? | Should be paid salary...................................1  Community helping in their farm…………………………….................2  Respect by community members…………3  Motivation/Incentives................................4  Provide logistics for the work…………….5  Provide bicycles/boots/rain coat………….6  Awards…………………………………...7  Other (specify)............................................8 | Q15ATRAC |

**SECTION 4: SELECTION, TRAINING, SUPERVISION AND PERFORMANCE**

| 16. | How are health volunteers selected? | By the chief/elders.........................................1  By the assembly person.................................2  Community members.....................................3  Community group leaders.............................4  Relatives/family members.............................5  Program officers/nurse……………..............6  By choice……………………………………7  Other (specify)………………………………8 | Q16SELEC |
| --- | --- | --- | --- |
| 17. | How were you selected? | By the chief/elders..........................................1  By the assembly person..................................2  Community members.....................................3  Community group leaders...............................4  Relatives/family member...............................5  Program officer/nurse………………………6  By choice……..……………………….........7  Other (specify)..............................................8 | Q17OWNSE |
| 18. | What qualities must one have before you are selected as a health volunteer?  **CIRCLE ALL THAT APPLY** | **Yes No**  Hard working person.......................1 2  Come from the community..............1 2  Understand the local language.........1 2  Have patience/respect for people.....1 2  Read and write.................................1 2  Have interest in the work.................1 2  ready to work without pay...............1 2  Trustworthy person………………..1 2  Others……………………………..1 2  (specify)......................................................... | Q18HWORK  Q18FRCOM  Q18ULANG  Q18PAT  Q18RWRIT  Q18INTER  Q18NPAY  Q18TRUST  Q18OTHER  Q18SPECI |
| 19. | Did you receive training when you were first recruited? | Yes...........................................................1  No............................................................2 | Q19TRAIN  **Q25** |
| 20. | How long were you trained? | Less than one week..................................1  One week.................................................2  Two weeks...............................................3  Three weeks.............................................4  Other (specify).........................................5  NA……………………………….…….88 | Q20HLONG |
| 21. | What were you trained on?  **CIRCLE ALL THAT APPLY** | **Yes No**  Health education strategy…………1 2  Identify/treat simple malaria……...1 2  Mobilize people for  health programs…………………….1 2  Counseling services……………...…1 2  How to weight children……….…...1 2  Other……………………………….1 2  (Specify)…………………………………….  NA…………………………………….88 | Q21HEDU  Q21TREAT  Q21MOBIL  Q21COUNS  Q21WEIGH  Q21OTHER  Q21SPECI |
| 22. | Do you think the training was adequate to help you do the work well? | Yes............................................................1  No...............................................................2  NA………………………………………88 | Q22TENGH  **Q24** |
| 23. | If No, why?  (please, write two reasons on the lines provided) | NA………………………………………..88 | Q23WHY |
| 24. | Where were you trained? | DHMT............................................................1  At the sub-district ……………......................2  Outside the district..........................................4  Other (specify)...............................................5  NA………………………………………….88 | Q24WHTRA |
| 25. | Are you supervised? | Yes............................................................1  No.............................................................2 | Q25SUPER  **Q30** |
| 26. | How often are you supervised? | Once a week..............................................1  Once in two weeks.....................................2  Once a month.............................................3  Twice a month...........................................4  Once every three months...........................5  Other (specify)..........................................6  NA……………………………………..88 | Q26OFSUP |
| 27. | Who supervises you? | No supervision……………………………..1  Community members...................................2  The Sub-district head....................................3  Someone from DHMT..................................4  Sub-district health staff………………….…5  Other (specify)……………………………...6  NA………………………………………...88 | Q27WHOSU |
| 28. | How will you grade the supervision your receive for your work | Very Effective……………………..………..1  Effective…………………………………….2  Somewhat effective…………………………3  Not-effective………………………………..4  NA…………………………………………88 | Q28GRADS |
| 29. | How does supervision help in the work that you do? | Helps me do the work well………………..1  Motivate me to work harder……….……...2  Makes me feel important…………….........3  Makes me more committed………………..4  Other (specify)…………………………....5  NA………………………………………..88 | Q29SUPHE |
| 30. | How often do you do your work as a health volunteer? | Daily……………………………………..1  Once every week………………….……..2  Twice a week…………………………….3  Once every two weeks…………………..4  Once every month……………………….5  Other (specify)…………………………..6 | Q30OFTWK |
| 31. | Do you give reports to your supervisor? | Yes…………………………………………..1  No…………………………………………...2 | Q31GREPO  **Q34** |
| 32 | How often are you supposed to submit reports to your supervisor? | Every week…………………..………………1  Every two weeks…………….……………….2  Every month…………………………............3  Every quarter………………………………..4  Other (specify)………………………………5  NA……………………………………..….88 | Q32OFSUB |
| 33. | How often are you able to submit your reports to your supervisor? | Always…………………………………..…1  Somehow………………………………..…2  Rarely…………………………….……..…3  NA……………………………………..…88 | Q33OFTSU |
| 34. | Are there regular meetings? | Yes………………………………………..…1  No………………………………………...….2 | Q34REGUM  **Q37** |
| 35. | How often do you meet? | Once every week………………….………....1  Once every two weeks………………………2  Once a month……….…………………….…3  Other (specify)………………………...........4  NA…………………………………………88 | Q35OFMET |
| 36. | How often are you able to attend these meetings? | Always………………………………….……1  Somehow……………………………….........2  Rarely……………………………..………....3  NA………………………………….............88 | Q36OFMET |
| 37. | Do you take part in immunization activities all the time? | All the time………………………….............1  Sometimes.……………………………..……2  Rarely…………………………………..……3  Not part of immunization activities……........4 | Q37IMMUZ |
| 38. | What benefits does the community get from your work?  **(Write three benefits on the lines)** |  | Q38BENEF |
| 39 | What are the factors that affect your performance in the work?  **CIRCLE ALL THAT APPLY** | **Yes No**  No salary……………………..…..1 2  No community support/  recognition……………………….1 2  Lack of motivation/incentive…….1 2  No enough training…....................1 2  No effective supervision…............1 2  Workload…………………...........1 2  No time…………………………..1 2  No logistics……………………….1 2  No means of transport……………1 2  Other……………………………..1 2  (specify)…................................................. | Q39NSALA  Q39NCSUP  Q39NMOTI  Q39NTRAN  Q39NESUP  Q30WLOAD  Q39NTIME  Q39NLOGI  Q39TRANS  Q39OTHER  Q39SPECI |
| 40. | What do you think if done will help you do the work well?  **CIRCLE ALL THAT APPLY** | **Yes No**  Should be paid salary….…..............1 2  Community support/recognition......1 2  Motivation/incentive………………1 2  Enough training……………………1 2  Effective supervision....…………....1 2  Reduce workload…………………..1 2  Provide means of transport………..1 2  Provide logistics…………………..1 2  Other……………………………....1 2  (specify)…..................................................... | Q40PSALA  Q40CSUPO  Q39MOTI  Q40ETRAI  Q40ESUPE  Q40RWORD  Q40PTRAN  Q40LOGIS  Q40OTHER  Q40SPECI |

**SECTION 5: RETENTION AND SUSTAINABILITY OF CBHVs ACTIVITIES**

| 41. | Are people sometimes selected and trained as volunteers and later leave the work? | Yes…..........................................................1  No…...........................................................2 | Q41VLIVE  **Q45** |
| --- | --- | --- | --- |
| 42. | Do you know of any volunteer who has left the job? | Yes……………………………………..…1  No………………………………………..2  NA………………………………….…..88 | Q42KLIVE |
| 43. | In the past two years, how many volunteers do you know have left the job in this sub-district? | \|  \|  \| \| --- \| --- \|   NA……………………………………….88 | Q43NLIVE |
| 44. | What in your opinion makes them leave?  **Circle all that apply** | **Yes No**  No salary…………………………...1 2  No comm. support/recognition …...1 2  Old age…………...….......................1 2  No motivation/incentive…………...1 2  Illness/death…..................................1 2  No enough training….......................1 2  No effective supervision…...............1 2  Work is difficult/workload...............1 2  Got job elsewhere…….....................1 2  No time…………………………..…1 2  Other………………………………..1 2  (specify)…...................................................  NA…………………………………….88 | Q44NSALA  Q44NCSUP  Q44OAGE  Q44NMOTI  Q44ILL  Q44NTRAN  Q44NESUP  Q44WLOAD  Q44GJOB  Q44NTIME  Q44OTHER  Q44SPECI |
| 45. | What in your opinion affects or influences sustainability of CBHVs activities in this sub-district?  **Circle all that apply** | **Yes No**  No salary…………………………….1 2  No motivation/incentives....................1 2  No community support/recognition....1 2  No effective supervision.....................1 2  No enough training……....................1 2  No com. Involvement in activities…..1 2  No family/spouse support….………..1 2  Got job elsewhere……………...……1 2  Other ………………………………..1 2  (specify)…..................................................... | Q45NSALA  Q45NMOTI  Q45NCSUP  Q45NESUP  Q45NETRA  Q45NCINV  Q45NFSUP  Q45GJOB  Q45OTHER  Q45SPECI |
| 46 | What do you think should be done to retain CBHV?  **Circle all that apply** | **Yes No**  Should be paid salary….……............1 2  Com. support/recognition..................1 2  Community involvement in  program activities…..........................1 2  Motivation/incentives…...………….1 2  Give Awards………………………..1 2  Means of transport………………….1 2  Other………………………………..1 2  (specify)…….................................................... | Q46PSALA  Q46CSUPP  Q46CINVO  Q46MOTIV  Q46AWARD  Q46TRANS  246OTHER  Q46SPECI |
| 47. | Name of sub-district | Wuru……………………………………….1  Kologo……………………………………..2  Pungu………………………………………3  Paga…………………………………….….4  Navio/Nakolo……………………………..5  Mirigu/Nabanbo………………………..…6 | Q47NSUBD |

**THANK YOU FOR YOUR TIME!!!**
